# Gesundheit von Inhaftierten im Jugendstrafvollzug – Forschungsbefunde und Einblick in die Versorgungspraxis

**DOI:** 10.1007/s00103-025-04151-0

**Published:** 2025-10-30

**Authors:** Stefan Suhling, Uwe Meinecke

**Affiliations:** 1 Kriminologischer Dienst, Bildungsinstitut des niedersächsischen Justizvollzuges, Fuhsestr. 30, 29221 Celle, Deutschland; 2 Institut für Psychologie, Universität Hildesheim, Hildesheim, Deutschland; 3 Jugendanstalt Hameln, Hameln, Deutschland

**Keywords:** Jugendstrafvollzug, Gesundheitsversorgung, Psychische Erkrankungen, Vollzugsabteilung mit psychiatrischem Schwerpunkt, Psychosoziale Belastungen, Juvenile prison, Health care, Mental disorder, Psychiatric ward, Detention, Psychosocial stress

## Abstract

Junge Menschen im Gefängnis weisen im Vergleich zu Gleichaltrigen eine ausgeprägte Häufung biografischer und psychosozialer Belastungen auf, insbesondere ein niedriges Bildungsniveau und multiple Gewalterfahrungen. Diese Belastungen spiegeln sich nicht nur in teilweise gravierenden Straftaten wider, sondern auch in einem überdurchschnittlichen Substanzmittelkonsum und einer deutlich erhöhten Prävalenz psychischer Auffälligkeiten und Störungen. Der Artikel skizziert zunächst psychosoziale Belastungsfaktoren im Zusammenhang mit körperlichen und psychischen Gesundheitsproblemen und beschreibt anschließend die gesundheitliche Versorgung im Jugendstrafvollzug.

Über die körperlichen und psychischen Erkrankungen inhaftierter Jugendlicher in Deutschland liegen kaum systematische und aktuelle Studien vor. Ausnahmen stellen die Bereiche Substanzmittelmissbrauch und Suizidalität dar. Ebenso besteht ein Mangel an groß angelegten Untersuchungen zur medizinischen sowie psychiatrisch-psychologischen Versorgung im Jugendstrafvollzug. Daraus ergibt sich ein erheblicher Forschungsbedarf hinsichtlich der gesundheitlichen Situation junger Inhaftierter und ihrer Versorgungspraxis. Studien legen nahe, dass für eine adäquate medizinische und psychologisch-psychiatrische Betreuung im Jugendstrafvollzug ein höherer Ressourceneinsatz nötig ist.

## Abstract (en)

Compared to their peers, young people in prison show a marked accumulation of biographical and psychosocial stressors, in particular a low level of education and multiple experiences of violence. These stressors are reflected not only in sometimes serious criminal offences, but also in above-average substance use and a significantly increased prevalence of mental health issues and disorders. The article first outlines psychosocial stress factors in connection with physical and mental health problems and then describes healthcare in the juvenile justice system.

There are hardly any systematic and up-to-date studies on the physical and mental illnesses of incarcerated young people in Germany. The areas of substance abuse and suicidality are exceptions. There is also a lack of large-scale studies on medical and psychological-psychiatric care in juvenile detention. This results in a considerable need for research on the health situation of young prisoners and their care practices. Studies suggest that adequate medical and psychological-psychiatric care in juvenile detention needs more resources.

## Hintergrund

Jugendliche (also 14- bis 17-Jährige) und Heranwachsende (18- bis 20-Jährige), die eine Jugendstrafe gemäß § 17f. Jugendgerichtsgesetz (JGG) zu verbüßen haben, gelangen in Deutschland in den Jugendstrafvollzug. Auch Personen, die in diesem Alter in Untersuchungshaft genommen werden, sowie Jungerwachsene zwischen 21 und 24 Jahren, die zu einer Freiheitsstrafe verurteilt wurden, aber noch für den Jugendstrafvollzug geeignet sind, können dort untergebracht werden (§ 114 JGG). Andererseits können auch heranwachsende Gefangene aus dem Jugendvollzug gem. § 89b JGG ausgenommen und in den Strafvollzug für Erwachsene verlegt werden, wo für sie dann das Strafvollzugsgesetz für Erwachsene gilt.

Jugendstrafanstalten, mitunter, wenn auch etwas irreführend, als „Jugendanstalten“ bezeichnet, sind spezielle Einrichtungen des Justizvollzugs, die eigenen gesetzlichen Regelungen unterliegen [1]. Aufgrund des im Vordergrund stehenden Resozialisierungs- bzw. Erziehungsziels sind sie personell meist besser ausgestattet als der Erwachsenenstrafvollzug und verfügen über ein spezifisches Maßnahmenangebot, das auf die besonderen Bedarfe junger Inhaftierter zugeschnitten ist. In Deutschland gibt es 26 Anstalten, die ausschließlich oder auch Jugendstrafe vollziehen. Am 30.11.2024 befanden sich 2582 junge Männer und 108 junge Frauen zwischen 14 und 24 Jahren im Jugendstrafvollzug [2]. 16 % von ihnen waren Jugendliche. Hinzu kommen 1353 Untersuchungsgefangene bis inklusive 20 Jahre; wie viele der 1975 21–24-jährigen Untersuchungsgefangenen in Jugendanstalten einsaßen, ist den Zahlen nicht zu entnehmen.

Bezieht man sich auf die 2690 Personen, die im Jugendstrafvollzug eine Jugend- oder Freiheitsstrafe verbüßten, so machten sie nur 6,4 % der insgesamt 42.308 Personen aus, die insgesamt eine Freiheits- oder Jugendstrafe absaßen. Der Anteil der bis inkl. 24 Jahre alten Personen an allen Tatverdächtigen des Jahres 2024 betrug indes 30,7 % [3]. Die Gefangenenzahl^1^ für die bis 24 Jahre alten Personen betrug 2024 32,3, die für ältere 61,6. Die Tatverdächtigenbelastungszahlen^2^ betrugen im gleichen Jahr indes 5711 für Jugendliche, 5447 für Heranwachsende und 4782 für Jungerwachsene, während sie für alle Personen über 21 nur 2199 betrug. Solche Relationen zeigen, dass einer deutlichen *Über*repräsentation junger Menschen auf der Tatverdächtigenebene eine deutliche *Unter*repräsentation bei den Gefangenen gegenübersteht. Dies liegt nicht nur an der durchschnittlich geringeren Tatschwere bei jungen Menschen, sondern auch an der Zurückhaltung der Jugendgerichte bei der Verhängung von Gefängnisstrafen auf der Basis des JGG.

Aus diesen Zahlen bzw. Relationen lässt sich die Vermutung ableiten, dass es sich bei der Population junger Gefangener um eine stark selektierte Personengruppe handelt, in der man eine weite Verbreitung verschiedener psychosozialer Problemlagen erwarten kann. Auch lässt sich annehmen, dass die Gruppe eine Negativauswahl im Hinblick auf gesundheitliche Beeinträchtigungen, insbesondere psychische Störungen, darstellt.

Diesen Vermutungen soll im Folgenden in Form eines narrativen Reviews nachgegangen werden. Grundlage bildet in erster Linie eine Analyse der einschlägigen Literatur mit Bezug zu Deutschland sowie – soweit relevant – zu Österreich und der Schweiz. Sofern keine entsprechenden Publikationen verfügbar sind, werden die Ergebnisse aktueller internationaler Reviews und Metastudien herangezogen; internationale Einzelstudien werden nicht berücksichtigt. Eine weitere Eingrenzung bezieht sich auf die Zielgruppe der jungen Inhaftierten; Arbeiten zu erwachsenen Gefangenen werden nicht einbezogen.

Im Folgenden werden zunächst kurz aktuelle Befunde zu psychosozialen Belastungsfaktoren bei Jugendstrafgefangenen rekapituliert, die im Kontext der physischen und vor allem psychischen gesundheitlichen Herausforderungen von Bedeutung sind. Anschließend wird auf die körperliche und psychische Gesundheit von jungen Gefangenen eingegangen. Darauf folgt eine Betrachtung der Gesundheitsversorgung im Jugendstrafvollzug. In einem Exkurs wird zudem eine kurze Fallschilderung durch den Zweitautor vorgestellt, der als Psychiater in der Jugendanstalt Hameln (Niedersachsen) tätig ist. Er versorgt dort Gefangene ambulant und hat zusammen mit anderen Bediensteten eine Vollzugsabteilung mit psychiatrischem Schwerpunkt aufgebaut. Abschließend werden Schlussfolgerungen abgeleitet.

## Psychosoziale Merkmale von Inhaftierten im Jugendstrafvollzug

Inhaftierte im Jugendstrafvollzug (im Folgenden: JSG – Jugendstrafgefangene) stellen die Institution und letztlich auch die Gesellschaft aufgrund ihrer multiplen Problemlagen vor Herausforderungen. Diese Feststellung trifft zwar auch auf erwachsene Inhaftierte zu; die oben beschriebenen Selektionsmechanismen der Strafverfolgung führen aber dazu, dass sie für junge Menschen im Gefängnis besonders relevant ist (zur Übersicht [4–6]). Tab. 1 gibt einen Überblick über ausgewählte Merkmale.

**Tab. 1:** Psychosoziale Problemlagen von Jugendstrafgefangenen nach verschiedenen Quellen

Problemlage	Ausprägung	Anteile (Stichprobengröße) nach verschiedenen Quellen
Geringer Bildungsgrad	Ohne Schulabschluss	50,1 % (4615)^a^
		54,0 % (535)^b^
		63,7 % (6266)^c^
	Ohne Berufsabschluss	94,2 % (4615)^a^
		97,0 % (535)^b^
		97,3 % (6266)^c^
Unzureichende Deutschkenntnisse	Bei im Ausland geborenen Jugendstrafgefangenen ohne deutsche Staatsangehörigkeit	55,0 % (268)^d^
Physische Gewalterfahrungen in der Biografie	Durch die Eltern	45,3 % (865)^e^
	In der Schulzeit	51,8 % (865)^e^
Physische Gewalt als Anlassdelikt	–	61,1 % (535)^b^
	–	65,8 % (6266)^c^
Physische Gewalt in der Haft	„In den letzten vier Wochen“	Begangen: 33,0 % (865)^e^
		Erfahren: 34,1 % (865)^e^
	„In den letzten drei Monaten“	Begangen: 62,0–68,0 % (882)^f^
Sanktionsbelastung im Vorfeld der Inhaftierung	Vorherige Sanktionen (ohne Diversionsentscheidungen)	Mittelwert 2,8 (535)^b^
	Eintragungen im Bundeszentralregister	Mittelwert 4,0 (241)^g^
	Vorherige Bewährungsstrafe	73,1 % (275)^h^
Wiederverurteilung innerhalb von 3 Jahren nach Entlassung	–	62,0 % (im Vergleich zu 43,0 % der aus dem Erwachsenen-Strafvollzug Entlassenen)^i^

Quellen:

^a^ Bayerischer Jugendstrafvollzug [7]

^b^ Jugendstrafvollzug Baden-Württemberg [8]

^c^ Diverse Bundesländer (Berlin, Brandenburg, Bremen, Hessen, Nordrhein-Westfalen, Sachsen, Sachsen-Anhalt) [9]

^d^ [10]

^e^ [11]

^f^ [12]

^g^ [13]

^h^ [14]

^i^ [15]

So zeigen deutsche Forschungsergebnisse, dass JSG über eine sehr geringe Bildung in Form von Schul- oder Berufsabschlüssen verfügen und dass ein nicht geringer Teil der ausländischen Inhaftierten nur unzureichend Deutsch spricht. Die Gewaltbelastung von JSG ist in mehrfacher Hinsicht hoch: Sie haben häufig im Elternhaus und in der Schule körperliche Gewalt erlebt, mehr als die Hälfte von ihnen ist zur aktuellen Jugendstrafe wegen eines Gewaltdelikts (meist Raub oder Körperverletzung) verurteilt worden. Auch in der Haft setzen sich Opfererfahrungen und Täterhandeln in Bezug auf Gewalt vielfach fort, wobei viele sowohl Gewalt ausüben als auch erleiden [12]. Die durchschnittliche Sanktionsbelastung vor der Anlasstat war bereits hoch; fast nie ist die Anlasstat die erste abgeurteilte Tat gewesen. Auch der Anteil derjenigen, die innerhalb von 3 Jahren nach der Entlassung erneut verurteilt werden, liegt bei über 60 %.

Aus den Ergebnissen lässt sich ableiten, dass bei JSG ein hoher Bedarf an Behandlungsmaßnahmen diagnostiziert wird: 55,7 % sollen an sozialen Trainingsmaßnahmen, 38,1 % an einer Antigewaltmaßnahme teilnehmen, bei 8,9 % wird eine Sozialtherapie empfohlen, 39,1 % sollen Schuldnerberatung/Schuldenregulierung in Anspruch nehmen [16].

Weitere Untersuchungen erfassten Behandlungsbedarfe und Risiken auf Ratingskalen im Rahmen der Anfangsdiagnostik im Jugendstrafvollzug. Hier zeigte sich z. B. bei 37 % das Risiko einer Gewaltbereitschaft, nur bei ca. 33 % waren realistische Zukunftspläne festzustellen; nicht einmal die Hälfte arbeitete aktiv an der Erreichung des Vollzugsziels mit; weniger als 3 von 10 setzten sich ernsthaft mit ihrer Straftat auseinander [16]. Veröffentlichungen über eine Befragungsstudie mit mehreren Hundert JSG identifizierten neben Schulproblemen dissoziale Einstellungen/Kognitionen und dissoziale Persönlichkeitsmerkmale wie geringe Emotionsregulation und Selbstkontrolle als rückfallrelevante Problemfelder [14].

Erfahrungsberichten aus der Praxis zufolge gibt es weitere Belastungen: Viele JSG seien zuvor in der Kinder- und Jugendpsychiatrie gewesen, in Heimen und Pflegefamilien aufgewachsen, waren vor der Inhaftierung obdachlos oder hätten sich in vielen europäischen und außereuropäischen Ländern in Flüchtlingsheimen und Gefängnissen aufgehalten. Heute stammten viele aus Kriegsgebieten, wo sie oftmals psychischen und physischen Gewalterfahrungen ausgesetzt gewesen seien (vgl. zu einzelnen dieser Aspekte auch [4, 13]).

Zusammengenommen zeichnen die Ergebnisse zu psychosozialen Belastungsfaktoren bei JSG ein recht ausgeprägtes Profil an Problemen in den Bereichen Biografie, Bildung, Gewalt, Denken und Verhalten. Diese Bereiche können im Kontext der in diesem Beitrag im Fokus stehenden physischen und vor allem psychischen gesundheitlichen Herausforderungen nicht außer Acht gelassen werden, da sie gemeinsam als Ausprägungen eines bestimmten, abweichenden und mitunter dissozialen Lebensstils begriffen werden können, der viele Personen im Jugendstrafvollzug vor der Inhaftierung kennzeichnete. Daneben gibt es seriöse Belege für Zusammenhänge zwischen belastenden Kindheitserlebnissen (Adverse Childhood Experiences – ACE) auf der einen und psychischen Störungen, Aggression, Impulsivität, Substanzmissbrauch und Delinquenz [17] sowie auch körperlichen Erkrankungen [18] auf der anderen Seite.

## Gesundheitliche Probleme von Inhaftierten im Jugendstrafvollzug

Während über die psychosozialen Merkmale der Insassen vergleichsweise viele Erkenntnisse für den deutschen Jugendstrafvollzug vorliegen, ist die Datenlage hinsichtlich physischer und psychischer Probleme und Erkrankungen eher spärlich. Besser ist sie in Bezug auf missbräuchlichen Substanzmittelkonsum und Suizid.

### Körperliche Erkrankungen

Deutsche Prävalenzstudien zu körperlichen Erkrankungen speziell im Jugendstrafvollzug sind den Autoren nicht bekannt. Erfahrungsberichte haben Handke und Lehmann (2009) sowie Schindler (2014) vorgelegt [19, 20]. Danach lässt sich die Gesundheit von JSG kennzeichnen durch fehlende oder lückenhafte Kinder- und Jugendvorsorgeuntersuchungen, fehlenden Impfschutz, „ruinösen Zahnstatus“ [19, S. 139]. Die allgemeinen gesundheitlichen Probleme Jugendlicher (Schäden am Muskel-Skelett-System, Diabetes, Übergewicht, Bluthochdruck, Asthma, Allergien) kämen im Jugendstrafvollzug besonders häufig vor, zumal viele aus ärmeren Verhältnissen stammten und ein enger (negativer) Zusammenhang zwischen Armut und Gesundheitszustand bestehe.

Eine Übersichtsarbeit über weltweite Studien zu Gesundheit und Krankheit junger Menschen im Gefängnis liegt von Borschmann et al. aus dem Jahr 2020 vor [21]. Sie stellt eine hohe Lebenszeitprävalenz von physischen und psychischen Gesundheitsproblemen, -risiken und -bedingungen fest. JSG seien hiervon stärker belastet als ihre nichtinhaftierten Peers. In Bezug auf körperliche Erkrankungen würden neben Substanzmittelkonsumstörungen vor allem durch Blut übertragene Krankheiten und sexuell übertragbare Krankheiten bei JSG gehäuft vorkommen, wie z. B. Chlamydien, Syphilis, Hepatitis C und HIV. Die Prävalenz für Syphilis liegt demnach zum Beispiel unter den Inhaftierten im Median bei 2,8 % (Interquartilsabstand 0,9–3,4 %), während sie in den USA in der adoleszenten Allgemeinbevölkerung unter 0,01 % liegt. Für Gonorrhö liegt sie unter Gefangenen in den USA bei 1,7–2,0 % im Vergleich zu 0,4 % in der dortigen altersentsprechenden Bevölkerung. Ein Review US-amerikanischer Studien von 2022 bestätigte die Befunde zur erhöhten Prävalenz sexuell übertragbarer Krankheiten auch unter jungen Inhaftierten [22]. Eine internationale Übersichtsarbeit zu Schädel-Hirn-Traumata zeigt auf, dass junge Inhaftierte diese häufiger erlebt hatten als gleichaltrige Nichtinhaftierte und dass die Diskrepanz in der Prävalenz mit der Schwere des Traumas steigt [23]. Die Übertragbarkeit der Befunde auf Deutschland ist unklar bis fraglich.

Ein wichtiger Anlass für eine medizinische Behandlung im Jugendstrafvollzug kann eine körperliche Auseinandersetzung bzw. Gewalterfahrung sein. Physische Gewalt und damit körperliche Verletzungen kommen unter JSG häufiger vor als unter erwachsenen Inhaftierten [11]. Kosenko und Steger [24] analysierten medizinische Akten aus dem Jugendstrafvollzug der DDR; ihnen fiel unter anderem die Häufung solcher Behandlungsanlässe auf.

### Neurologische und psychische Störungen

Die Arbeit von Borschmann et al. widmete sich auch neurologischen Entwicklungsstörungen [21]. Den Ergebnissen zufolge leiden junge Inhaftierte im Vergleich zu ihren nichtinhaftierten Peers gehäuft unter einer Aufmerksamkeitsdefizit‑/Hyperaktivitätsstörung (ADHS), Kommunikationsstörungen, einer fetalen Alkoholspektrumstörung, Lernbehinderungen und traumatischen Gehirnschädigungen. Während die Prävalenz für die fetale Alkoholspektrumstörung zum Beispiel unter Inhaftierten auf 11–21 % geschätzt wird, wird für die adoleszente Allgemeinbevölkerung ein Anteil von 5–7 % angenommen. Überrepräsentiert in unterschiedlichem Ausmaß seien junge Inhaftierte auch im Hinblick auf Gemütserkrankungen, Major Depression, Angststörungen, posttraumatische Belastungsstörungen und schizophrene Störungen. Störungen mit oppositionellem Trotzverhalten und Störungen des Sozialverhaltens waren deutlich häufiger unter JSG zu finden als unter nichtinhaftierten Jugendlichen (für die Störung des Sozialverhaltens z. B. 73,5 % (Konfidenzintervall 53,9–79,8 %) unter Inhaftierten vs. 2,1 % in der jungen Gesamtbevölkerung). Dies korrespondiert mit den oben berichteten Ergebnissen zum devianten (normabweichenden) Lebensstil.

Kurze Zeit später bestätigte eine systematische Übersichtsarbeit von Beaudry et al. die hohen Prävalenzraten psychischer Störungen bei jungen Inhaftierten sowie das deutlich häufigere Vorkommen bei Inhaftierten im Vergleich zur gleichaltrigen Allgemeinbevölkerung [25]. So betrage die Prävalenz für psychotische Erkrankungen 2,7 % (Konfidenzintervall 2,0–3,4 %), dies sei ungefähr das 10-Fache der Verbreitung unter Gleichaltrigen in der Allgemeinbevölkerung. Ungefähr eine von 5 jungen Personen im Strafvollzug leide an einer ADHS, unter nichtinhaftierten jungen Menschen sei nur eine von 10 betroffen. In der genannten Arbeit werden auch Unterschiede zwischen weiblichen und männlichen JSG im Hinblick auf die psychischen Störungen berichtet; so weisen Erstere höhere Raten bei Major Depression und posttraumatischer Belastungsstörung auf. Ein Überblickstext von Fovet et al. (2024) weist darauf hin, dass die Prävalenzraten psychischer und neurokognitiver Störungen unter jungen Inhaftierten auch höher seien als unter erwachsenen männlichen Gefangenen [26].

Deutsche Arbeiten zu psychischen Störungen unter JSG sind selten. Im Jahr 2003 fand im offenen Jugendstrafvollzug in Niedersachsen eine Untersuchung mit 40 JSG statt [27]. Die Störungsdiagnostik wurde mittels der strukturierten klinischen Interviews für das DSM-IV durchgeführt. 31 der 40 JSG erhielten mindestens eine Diagnose auf Achse I für die Lebenszeitprävalenz (27,5 % affektive Störungen, 12,5 % Angststörungen, 62,5 % substanzbezogene Störungen). Bei 65 % der im Durchschnitt etwas über 20-Jährigen wurden auch eine oder mehrere Persönlichkeitsstörungen gefunden, zumeist die antisoziale. Eine Studie in Schleswig-Holstein [28] mit Neuzugängen in die Jugendstrafanstalt (*N* = 149) ergab nichtsubstanzbezogene Achse-I-Prävalenzen von 81 % für Störungen des Sozialverhaltens, 6,7 % für Angststörungen, 6 % für Depressionen, 4,1 % für psychotische Störungen und 0,7 % für Essstörungen. Bei 16,0 % wurde eine Borderline- und bei 11,4 % eine narzisstische Persönlichkeitsstörung festgestellt. Grieger berichtet über eine Prävalenz von ADHS, die in ihrer nichtklinisch diagnostizierten Stichprobe (*N* = 283) bei 19,4 % lag [14]. Köhler et al. zeigten in einer eigenen, narrativen Literaturübersichtsarbeit, dass junge Gefangene in allen Störungskategorien im Vergleich mit der Allgemeinbevölkerung überrepräsentiert sind [29].

Seit Mitte der 2010er-Jahre wird in allen Bundesländern jährlich am 31.03. u. a. erfasst, wie viele Inhaftierte vor der Haft welche Substanzen missbräuchlich konsumiert bzw. von welchen Substanzen sie abhängig gewesen waren. Allerdings werden die Daten nicht in allen Ländern systematisch durch Ärztinnen und Ärzte erhoben. Stoll und Kollegen (2021) berichten für den Stichtag 31.03.2018, dass bei 29 % der JSG Substanzmissbrauch und bei 27 % Substanzabhängigkeit festgestellt wurde [30]. Die Gesamtprävalenz problematischen Konsumverhaltens lag damit über der anderer Inhaftiertengruppen. Eine aktuelle Auswertung für Niedersachsen (31.03.2025) weist für die Inhaftierten der Jugendanstalt 50 % Missbrauch und 8 % Abhängigkeit aus (Barkowski und Häßler, 2025, nicht veröffentlichter Beitrag des Kriminologischen Dienstes Niedersachsen). Wie in anderen Jahren auch, erwiesen sich Cannabinoide, gefolgt von multiplem Substanzgebrauch, als wichtigste Substanzgruppe. Der multiple Substanzkonsum dominierte hingegen bei den erwachsenen Inhaftierten mit Drogenproblemen. Die hohen Prävalenzraten werden in anderen, stichprobenbasierten deutschen Studien bestätigt [8, 14, 31]. Internationale Ergebnisse bestätigen die hohen Prävalenzen [32]. Nach Ergebnissen einer groß angelegten Dunkelfeldstudie in Deutschland konsumieren ca. 30 % der JSG auch in Haft Drogen, meist ebenfalls Cannabinoide [33].

### Suizidalität

Ein besonderes Problem im Kontext des Strafvollzugs stellt Suizidalität dar. So gingen in der Untersuchung aller Todesfälle im Hamburger Justizvollzug zwischen 1995 und 2012 durch Petersen et al. [34] immerhin 50 (von 130) auf einen Suizid zurück. Radeloff und Kollegen [35] ermittelten anhand einer Totalerhebung aller Suizide in deutschen Justizvollzugsanstalten zwischen 2000 und 2010 erhöhte Suizidraten unter Gefangenen im Vergleich zur Allgemeinbevölkerung. Dieser Befund war besonders ausgeprägt unter jugendlichen Inhaftierten, sie hatten eine 23-mal so hohe Suizidrate wie nichtinhaftierte Jugendliche. In einer Untersuchung in sächsischen Justizvollzugsanstalten, in die auch JSG einbezogen wurden, gaben ca. die Hälfte der 113 Befragten an, schon einmal in ihrem Leben suizidale Gedanken gehabt oder suizidale Handlungen ausgeführt zu haben [36]. Die internationale Metaanalyse von Borschmann et al. [21] bestätigt das erhöhte Suizidrisiko im Jugendstrafvollzug sowie eine erhöhte Prävalenz von Suizidversuchen und Suizidfantasien im Lebensverlauf. Eine aktuelle Metaanalyse von Barker et al. [37] weist auch auf ein erhöhtes Risiko von nichtsuizidalen selbstverletzenden Handlungen in dieser Gruppe hin.

Personale und biografische Risikofaktoren für Suizidalität kommen unter Jugendstrafgefangenen gehäuft vor [38]. Persönlichkeitsauffälligkeiten und -störungen etwa gingen eher mit Suizidalität einher [36], emotionale Probleme und eine Borderline-Persönlichkeitsproblematik korrelierten mit der Lebenszeitprävalenz selbstverletzenden Verhaltens [39]. Nichtdeutsche sind unter den Suizidentinnen und Suizidenten weder über- noch unterrepräsentiert [40]. Für männliche und weibliche junge Inhaftierte könnten unterschiedliche Risikofaktoren relevant sein [41]. Daneben wirken auch haftbezogene Faktoren (wie Autonomieverluste, Isolation, Unsicherheit, Gewalterfahrungen) risikoerhöhend, weil sie z. B. zu Anpassungsproblemen oder reaktiv-depressiven Reaktionen führen können. Zusammen genommen erscheinen Interaktionen zwischen personalen und gefängnisbezogenen Risikofaktoren zu bestehen, die das Suizidrisiko bei (jungen) Inhaftierten erhöhen [42].

## Gesundheitliche Versorgung im Jugendstrafvollzug

Psychisch beeinträchtigte oder gestörte JSG stellen das Gefängnis nicht nur wegen der berichteten erhöhten Wahrscheinlichkeit von Selbstverletzung und Suizidalität vor Herausforderungen. Auch regelverletzendes und gewalttätiges Verhalten kommen bei ihnen im Vergleich zu psychisch gesunden Inhaftierten gehäuft vor (s. [43] mit weiteren Nachweisen). Nicht nur für die Betroffenen selbst, sondern auch für die Mitinhaftierten und die Bediensteten stellt das Verhalten der psychisch beeinträchtigten Inhaftierten deshalb eine Belastung dar. Daneben gibt es Hinweise darauf, dass das Vorliegen einer psychischen Störung das Rückfallrisiko erhöht – zumindest dann, wenn es sich um eine externalisierende Störung handelt [44]. Insofern gefährden psychische Störungen auch die Erreichung des Vollzugsziels.

Die Inhaftierung selbst kann auch körperliche und psychische Belastungen mit sich bringen. Die Trennung von Angehörigen und Peers, starke Einschränkungen der Autonomie, die Verfügbarkeit von (im Vergleich zu „draußen“ deutlich teureren) Drogen und die Gefahr der Verschuldung, Unterdrückung und Viktimisierung durch Mitgefangene sind hier nur einige Beispiele. Internationale Studien zeigen, dass gerade zu Beginn der Inhaftierung das Belastungserleben steigt [45]. Möglicherweise bergen auch die offenbar gehäuft vorhandenen, durch Blut übertragbaren Erkrankungen Risiken – nicht nur für Bedienstete [46], sondern auch für die Mitgefangenen. Junge Menschen können insofern in mehrfacher Hinsicht als Personenkreis mit hohem psychosozialen und medizinischen Betreuungsbedarf betrachtet werden [47].

Umso wichtiger erscheint eine angemessene medizinische und psychologisch-psychiatrische Ressourcenausstattung im Jugendstrafvollzug. Die Inhaftierung kann nicht nur als Belastung, sondern auch als Chance begriffen werden, „mitgebrachte“ gesundheitliche Erkrankungen zu behandeln, Suchtprobleme durch Entgiftung oder auch Substitution in Kombination mit Beratung und Betreuung in den Griff zu bekommen und eine psychische Stabilisierung zu erreichen. Dadurch, dass die Inhaftierten sich, anders als in Freiheit, nicht entziehen können, besteht bei ausreichenden personellen und finanziellen Mitteln und angemessener Kontaktgestaltung die Gelegenheit, eine gründliche Diagnostik vorzunehmen, Behandlungsmaßnahmen – zum Teil in Zusammenarbeit mit den Bediensteten außerhalb des medizinischen Fachbereichs und mit externen Fachärztinnen und -ärzten – durchzuführen und auch die Versorgung nach der Entlassung anzubahnen. Über die multiplen Herausforderungen, mit der sich die Vollzugsmedizin dabei konfrontiert sieht, informieren z. B. der Sammelband von Keppler et al. [48] und die anderen Beiträge zum vorliegenden Themenheft. Die Beijing Rules der Vereinten Nationen (UN; [49]), die für die als besonders vulnerabel im Hinblick auf eine Inhaftierung betrachtete Gruppe der jungen Menschen entwickelt wurden, sowie die Europäischen Strafvollzugsgrundsätze [50] und die Strafvollzugsgesetze der Bundesländer legen mit dem Äquivalenzprinzip fest, dass in Haft die gleichen Versorgungsstandards gelten sollen wie außerhalb der Gefängnismauern (vgl. ausführlicher [51, 52]).

Aktuelle Zahlen zur tatsächlichen medizinischen und psychologisch-psychiatrischen Versorgung im Jugendstrafvollzug liegen für Deutschland allerdings nicht vor. Aus einer Antwort der Bundesregierung auf eine Kleine Anfrage (Bundestagsdrucksache 20/14853) vom 03.02.2025 geht hervor, dass am 01.09.2023 insgesamt 236,6 Arbeitskraftanteile (AKA) festangestelltes Personal im Ärztlichen Dienst zur Verfügung standen und dass weitere 202,3 AKA durch Vertragsärztinnen und -ärzte (auch auf Stundenkontingentbasis) vorhanden waren. Unterstützt wurden diese durch 92,7 AKA in der Krankenpflege. In Bezug auf die Versorgung der mentalen Gesundheit sind auch die knapp 985 AKA im Psychologischen Dienst und im weiteren Sinne auch die 1888 AKA im Sozialen Dienst relevant. Eine Differenzierung nach Jugend- und Erwachsenenvollzug ist aber nicht verfügbar.

Eine Taskforce der Deutschen Gesellschaft für Psychiatrie und Psychotherapie, Psychosomatik und Nervenheilkunde (DGPPN) hat im Frühjahr 2024 in 176 deutschen JVAs eine Online-Umfrage zur psychiatrischen Versorgung durchgeführt. 137 Anstalten antworteten [53, 54]. Bislang sind nur ausgewählte Ergebnisse über Internetmeldungen bekannt. So seien ca. 46 Stellen für psychiatrische Fachkräfte vorgesehen, von denen allerdings viele unbesetzt seien. Die Ressourcen für die ambulante Behandlung der Inhaftierten reichten den Angaben der Anstalten zufolge nicht aus. Besonders die Kapazitäten für vollstationäre psychiatrische Behandlungen wurden als unzureichend angegeben. Zu ähnlichen Ergebnissen kommt auch der – nicht auf wissenschaftlichen Studien beruhende – aktuelle Bericht der Nationalen Stelle zur Verhütung von Folter [55]. Eine kürzlich durchgeführte Befragung allgemeinpsychiatrischer Kliniken förderte vielfältige Probleme zutage, die resultieren, wenn psychisch auffällige Inhaftierte angesichts der Unterversorgung in den JVAs außerhalb des Gefängnisses stationär aufgenommen werden müssen [56].

Auch zum Themenkomplex der psychiatrischen Versorgung liegen keine separaten Ergebnisse für den Jugendstrafvollzug vor; es kann aber angenommen werden, dass er ähnlich betroffen ist wie der restliche Vollzug. Aus diesem Grund wird im folgenden Exkurs anhand eines kurzen Fallbeispiels eine Praxislösung zur psychiatrischen Versorgung in der Jugendanstalt Hameln beschrieben.

## Fallbeispiel: Die Vollzugsabteilung mit psychiatrischem Schwerpunkt in der Jugendanstalt Hameln

Nach der Weiterbildung von Krankenpflegerinnen und -pflegern zur psychiatrischen Fachpflege sowie der Einstellung zweier Psychiater öffnete in der Jugendanstalt Hameln 2018 eine „Vollzugsabteilung mit psychiatrischem Schwerpunkt“ (VPS) mit 20 Haftplätzen. Sie verfügt über 3 kameraüberwachte Hafträume und einen besonders gesicherten Haftraum für Notfälle (auch aus der Gesamtanstalt).

Herr A. kommt als Verlegung aus der Abteilung für „noch zur Mitarbeit am Vollzugsziel zu motivierende Inhaftierte“ in die VPS. Der bereits in der vorherigen Abteilung verhängte 14-tägige Zimmerarrest als Disziplinarmaßnahme aufgrund einer positiven Urinkontrolle (UK) auf Cannabinoide wird fortgesetzt. Ein Joint kostet gewöhnlich ein Päckchen Tabak. Herr A. hat Schulden bei Mitinhaftierten, was er in der letzten Sprechstunde offen eingeräumt hat. Nun wird verständlich, warum Herr A. in den letzten Wochen immer seltener bei der Arbeit war, die er eigentlich sehr gerne macht. Dort hätte er auf Personen treffen können, die von seinen Schulden wissen. Zunächst hat er sich durch den Anstaltsarzt wiederholt krankschreiben lassen, dann ist er öfter einfach nicht mehr zur Arbeit gegangen. Jetzt droht die Ablösung von seinem Arbeitsplatz. Sein Vollzugsabteilungsleiter (VAL) hatte den Psychiater vor Kurzem angesprochen, da Herr A. kaum noch den Haftraum verlasse, auch nicht mehr zur täglichen Freistunde. Der Nachtdienst sei abends häufiger zu ihm gerufen worden, da er über Panikattacken mit Atemnot, Schweißausbrüchen und Herzrasen klagte und nach Medikamenten verlangte, die ihm ein Psychiater früher verschrieben habe; sein Asthmaspray habe dagegen nicht geholfen. Außerdem sei sein Haftraumtelefon gesperrt worden, so teilte mir der VAL mit, da es nicht nachvollziehbare externe Geldüberweisungen auf sein Telefonkonto in der Anstalt gegeben habe.

Herr A. ist verzweifelt, hat aber Vertrauen zum Anstaltspsychiater, den er schon von einem kurzen U‑Haft-Aufenthalt vor 2 Jahren kennt, als er 15-jährig zum ersten Mal inhaftiert war und sich wegen Schlafstörungen in der Sprechstunde gemeldet hatte. Ihm war damals geholfen worden. Er berichtet, dass er nun noch schlimmere Schlafstörungen und vor allem Panikattacken habe. Er traue sich kaum, in die Freistunde zu gehen, und wenn, dann nur mit Schutz des Gebäudes im Rücken, da er sofort, wenn es etwas lauter und voller auf dem Freistundenhof werde oder Insassen ihm nahekämen, keine Luft mehr bekomme und sein Herz zu rasen anfange. Dann würden wieder die Bilder in ihm auftauchen, wie er im letzten Herbst auf einer Gartenparty mit einem Messer „abgestochen“ worden und dann nach einer Notoperation erst im Krankenhaus wiedererwacht sei. Beim Blick in die Krankenakte findet sich der Vermerk des Anstaltsarztes bei der Aufnahmeuntersuchung: „S27.0 Traumatischer Pneumothorax“. Er berichtet, dass er seitdem nur noch habe einschlafen können, wenn er anfangs verordnete und dann illegal eingenommene Benzodiazepine oder Cannabis konsumiert habe. Er vermittelt glaubhaft den Eindruck, wirklich ärztliche Hilfe zu suchen. Damit unterscheidet er sich von den zahlreichen Insassen, die fast alle imperativ Clonazepam und Pregabalin verlangen, dazu eine „Geschichte“ erzählen und eine „schmerzende“ Narbe zeigen, da sie wissen, dass neben Angststörungen und Epilepsie auch neurogene Schmerzen zur Indikation des letztgenannten Medikaments zählen.

Herrn A. wurde die Aufnahme auf die VPS angeboten. Dies hatte er vor 3 Monaten zunächst abgelehnt, trotz oder sogar wegen der Intrusionen, des Hyperarousals und der Panik- und Schlafstörungen. Denn: Jeden Abend habe er in seinem Haftraum lange mit der Mutter telefoniert, was ihn beruhigt habe. In der VPS gibt es zurzeit nur Flurtelefone; aus diesem Grund konnte er sich nicht zur Aufnahme entschließen. Damals wurde ihm explizit nur für einen Monat Bedenkzeit zur Aufnahme auf die VPS Zopiclon verschrieben (was selten und nur kurz gemacht wird, meist bei Patienten mit nächtlichen Panikstörungen und posttraumatischen Belastungsstörungen (PTBS)). Damit habe er gut schlafen können, sei danach jedoch wieder auf Cannabis umgestiegen und habe nun noch mehr Schulden und Ängste. Deshalb traue er sich nicht mehr zur Arbeit, da der mitinhaftierte Dealer dort „Kumpel“ habe. Anfangs habe er die Joints umsonst bekommen, nun würde er unter Druck gesetzt zur illegalen Drogenbeschaffung durch seine Besuchskontakte, ansonsten würde es ihm sehr schlecht ergehen. Er habe Angst vor körperlichen Übergriffen.

Herr A. wurde zunächst für eine Woche krankgeschrieben und ihm wurden wieder für 2 Wochen – jetzt unter Aufsicht der VPS – eine schlafanstoßende beruhigende Medikation verschrieben. Unter dem Schutz der therapeutischen Begleitung in der VPS konnte Herr A. sich dazu durchringen, zusammen mit weiteren Inhaftierten eine umfassende Meldung zu machen, sodass der „mitinhaftierte Dealer und seine Kumpel“ auf die Abteilung für nicht mitarbeitsbereite Insassen verlegt und von der Arbeit ausgeschlossen wurden. Herr A. wurde zurück in seine Abteilung des Normalvollzugs verlegt, geht wieder regelmäßig arbeiten und wird durch die psychiatrische Ambulanz, die ebenfalls vom Anstaltspsychiater betrieben wird, weiter begleitet.

Anstatt sich im weiteren Haftverlauf dem subkulturellen Milieu anzuschließen und zum „Überleben“ zum regelmäßigen Drogenschmuggel instrumentalisieren zu lassen, führte ein kurzer Aufenthalt auf der VPS und die anschließende niederfrequente ambulante Begleitung dazu, dass sich Herr A. auf seine Resozialisierung und die angestrebten Vollzugsziele im weiteren Haftverlauf konzentrieren konnte.

## Schlussfolgerungen

Ausgangspunkt dieses Beitrags war die aufgrund der Überrepräsentation junger Menschen auf der Ebene der Straftaten und der gleichzeitigen Unterrepräsentation im Gefängnis begründete Vermutung, dass Inhaftierte im Jugendstrafvollzug von einer Vielzahl psychosozialer Belastungsfaktoren sowie körperlicher und psychischer Erkrankungen bzw. Störungen betroffen sein sollten und dass sich die damit einhergehenden Bedarfe auch in der personellen Ausstattung der medizinischen Fachbereiche der Jugendstrafvollzugsanstalten widerspiegeln.

Tatsächlich stellen Inhaftierte im Jugendstrafvollzug eine Klientel mit besonderen Betreuungs- und Behandlungsbedarfen dar. Gut belegt, auch in Deutschland, sind Probleme im Bereich der Bildung, problematischer Sozialisationsbedingungen und Gewaltbelastungen (sowohl auf Opfer- als auch Täterseite). Internationalen Studien zufolge sind sie häufiger von körperlichen und vor allem von psychischen Störungen betroffen als ihre Altersgenossen in der Allgemeinbevölkerung – auch jenseits von Störungen mit explizitem Bezug zur Dissozialität.

Systematische Prävalenzstudien zu körperlichen Erkrankungen von Jugendstrafgefangenen liegen aus Deutschland keine vor. Hier sollten zukünftige Forschungen ansetzen. Zwar bestehen hohe datenschutzrechtliche Hürden, aber zu denken wäre etwa an systematische Analysen der medizinischen Akten (die in einigen Bundesländern auch digital vorliegen). Die wenigen deutschen Untersuchungen zu psychischen Störungen sind querschnittlich angelegte Einzelstudien mit spezifischen Gruppen; systematische und längerfristig angelegte Erhebungen wie zu Substanzmittelkonsumproblemen und Suiziden gibt es vor allem in dieser Zielgruppe nicht. Die deutschen Studien wurden auch Anfang der 2000er-Jahre durchgeführt und können als veraltet gelten. Im Bereich der körperlichen und auch der psychischen Erkrankungen besteht also aktuell eine große Forschungslücke in Deutschland. An der recht hohen Prävalenz und der Überrepräsentation der Probleme in dieser Zielgruppe kann insgesamt, aber auch heute wohl kein Zweifel bestehen. Die weite Verbreitung von körperlicher Viktimisierung und eigener Gewaltausübung in Haft dürfte das Problem verschärfen. Dafür, dass die Zahl der psychisch gestörten Inhaftierten steigt, wie häufig aus der Praxis zu hören ist, gibt es indes mangels aktueller bzw. regelmäßiger Studien wie ausgeführt keine wissenschaftlichen Belege.

Kaum belastbare Daten sind aktuell zur Ausstattung der medizinischen Fachbereiche der Jugendstrafanstalten verfügbar. Zwar gibt es aus dem politischen Raum Informationen zu verfügbaren Arbeitskraftanteilen, aber diese geben weder Auskunft über die tatsächlich besetzten Stellen noch liegen sie für den Erwachsenen- und Jugendstrafvollzug differenziert vor. Zukünftige Forschung sollte sich nicht nur der Stellenausstattung widmen, sondern vor allem der Frage, in welchem Umfang vorhandene Gesundheitsbedarfe vom medizinischen Personal bedient werden können (zu methodischen Herausforderungen dabei siehe [57]).

Den vorliegenden Informationen aus der Praxis zufolge ist die Versorgung von psychisch gestörten Inhaftierten unzureichend [58] und das sollte auch für den Jugendstrafvollzug zutreffen. Schon bei der Identifikation von psychischen Störungen dürfte sich die Versorgungslücke bemerkbar machen [59], folglich auch bei der angemessenen Behandlung. Ein Problem dabei ist, dass psychisch auffällige oder kranke Inhaftierte bei fehlender psychiatrisch-psychotherapeutischer Versorgung oft nur noch durch Absonderung, u. a. in besonders gesicherten Hafträumen, „behandelt“ werden können (vgl. zu fachlich-psychiatrischen Richtlinien [60]). Der Fachkräftemangel im medizinisch-psychiatrischen sowie im psychotherapeutischen Bereich verschont auch den Strafvollzug nicht, obwohl sich die Landesjustizverwaltungen Mühe geben, eine ausreichende Versorgung zu gewährleisten [61]. Wo dies gelingt, können sich erfolgreiche Behandlungsstrukturen und -prozesse entwickeln, wie das Fallbeispiel aus der Jugendanstalt Hameln illustriert hat.

## Footnotes

1 Die Gefangenenzahl berechnet sich als die Zahl der Gefangenen pro 100.000 Personen ihrer Altersgruppe in der Bevölkerung. Für die Gefangenenzahl der über 24-jährigen wurde die Bevölkerung bis zum 90. Lebensjahr herangezogen.

2 Die Tatverdächtigenbelastungszahl berechnet sich analog zur Gefangenenzahl als die Zahl der Tatverdächtigen pro 100.000 der jeweiligen Bevölkerung.
